# The Role of Sensory Perception in Consumer Demand for Tinned Meat: A Contingent Valuation Study

**DOI:** 10.3390/foods10092185

**Published:** 2021-09-15

**Authors:** Daniel Vecchiato, Biancamaria Torquati, Sonia Venanzi, Tiziano Tempesta

**Affiliations:** 1 Department of Land, Environment, Agriculture and Forestry, University of Padova, 35020 Legnaro, PD, Italy; tiziano.tempesta@unipd.it; 2 Department of Agricultural, Food and Environmental Sciences, University of Perugia, 06121 Perugia, PG, Italy; bianca.torquati@unipg.it (B.T.); sonia.venanzi@gmail.com (S.V.)

**Keywords:** food, canned meat, double hurdle, consumer preferences, sensory preferences, taste

## Abstract

This study presents an analysis of consumer preferences for a new food product: Tinned Chianina meat. Respondents (*N* = 249) participated in a sensory test, where they were also asked to declare their willingness to pay (WTP) for the tasted product. The WTP data were collected after the sensory test by means of the contingent valuation method using a payment card elicitation format. Data were analysed with Cragg’s double-hurdle model to understand which factors influenced market participation (WTP > 0) and then the variables that influenced the declared WTP. According to our results, sensory perception played a key role in explaining both participation in the market and the magnitude of the expressed WTP. Moreover, we found that the sensory aspects have a different effect on the decision to participate in the market and on the magnitude of the expressed WTP. Smell and flavour are the most important in determining the probability of entering the market, while texture has the greatest impact on the declared WTP.

## 1. Introduction

### 1.1. Preamble and Study Motivation

Product innovation plays a fundamental role for companies operating in the food sector. Food products, like other goods, have a life cycle that causes their demand to undergo a phase of decline. The latter can determine the cessation of their production or alternatively may require significant innovations. Product innovation allows us to increase profits in two main ways: by increasing either sales or prices. Knowledge of the demand function is therefore fundamental because it allows identification of the market share that corresponds to each level of the sales price. However, the demand for food depends on a number of factors that must be carefully considered before placing a new food product (NFP) on the market.

As highlighted by Köster [1], to obtain an exhaustive model of the factors influencing the choice of food products, the results of six different research areas must be coordinated: intrinsic product characteristics (perception), biological and physiological factors, psychological factors, situational factors, sociocultural factors, and extrinsic product characteristics (expectations).

In essence, the decision for a consumer to buy a new product depends in the first instance on the intrinsic and extrinsic cues, credence attributes and price [2,3]. Through them, the consumer builds expectations about the quality of the product. The consumer compares the expected quality with that of other substitute goods and will decide to buy it if the quality–price ratio is better than alternative options. In this regard, however, expectations on the quality of a product do not depend only on rational behaviour but are rather influenced by unconscious and emotional factors relating both to the consumer personally and to the purchase context.

Once a product has been purchased and tasted, the consumer can assess whether it meets his or her expectations; if it does, he/she will likely buy it again in the future [4]. Only the repeated consumption of the product can change the consumer’s habits and therefore favour its success on the market. Ultimately, it can be deduced that the complexity of the factors influencing consumer choices makes it difficult to verify a priori the potential degree of success of an NFP.

Considering that the development of NFPs requires considerable resources, scholars have developed many approaches that can be useful to a priori assess their market success. Consumers’ preferences towards NFPs can be analysed by means of hypothetical markets and affective methods. Hypothetical markets can be grouped into two broad categories: experimental markets, such as auctions [5], and stated preference approaches, such as the contingent valuation method (CVM), conjoint analysis and discrete choice experiments (DCEs) [6]. These methods have mainly been used to analyse the effect of non-experience attributes and extrinsic cues. Experience attributes are usually valued using affective methods [7], where the product is assessed by a panel of consumers after they taste it. Various methods are used to express the degree of liking, with hedonic scaling being one of the most frequently employed [8]. The disjointed application of the two above-mentioned approaches (hypothetical markets and affective methods) for the valuation of NFPs could fail to correctly predict real consumers’ behaviour. In fact, hypothetical markets fail to properly account for experience attributes, while, conversely, affective methods fail to consider important non-experience attributes, such as the product price. As noted above, consumers’ choices depend on food taste as well as other relevant factors [1]. For example, an NFP could have excellent sensory characteristics, but it might be too expensive for the consumer and, as a consequence, it will fail on the real market despite receiving excellent appreciation in sensory tests.

### 1.2. Study Objectives and Original Aspects

Our study contributes to the stated preference methods literature presenting the analysis of consumer preferences for an NFP: tinned Chianina [9] meat (see Appendix A for a description of the characteristics of the Chianina breed). We applied the CVM, asking participants to take part in a sensory test where sensory preferences were assessed by means of a nine-point hedonic scale considering five aspects: (i) appearance; (ii) smell; (iii) flavour; (iv) texture; and (v) overall liking. The willingness to pay (WTP) data were collected after the sensory test. Data were analysed with Cragg’s double-hurdle model [10] to understand which factors influenced market participation (WTP > 0) and which variables influenced the declared WTP.

Hypothetical methods, such as CVM and DCE, do not usually consider sensory preferences. In this respect, our research has the following objectives:

1. to test whether sensory preferences affect consumers’ willingness to buy a product;
2. to test whether sensory preferences affect consumers’ willingness to pay (WTP) for the product once they decide to enter the market; and
3. to understand which sensory preferences are crucial in the market participation phase and which influence WTP.

To pursue these aims, the use of Cragg’s double-hurdle model is interesting because, when applied to study the potential purchase of an NFP it can allow us to simulate real markets when an NFP is launched. Producers can follow two main strategies to induce the consumer to buy a product. On the one hand, they can engage adequately on product communication, informing consumers about the characteristics of the good in order to attract them. On the other hand, they can attract consumers by reducing the budgetary constraints that may preclude the purchase and tasting of the product. With reference to the latter aspect, sellers often try to induce consumers to taste an NFP using various strategies [11]. For example, they can attract consumers by allowing them to taste the NFP for free in the store, or they can offer it for sale at a very low price, indicating that the price is heavily discounted. After tasting it, consumers decide whether to buy it again in the future. This choice, however, will be conditioned by the price at which the good will be offered for sale, which will be purchased only in the event that the price is lower than or equal to the WTP, that is, the utility expected from consumption.

The model estimated with the double-hurdle procedure therefore provides two useful indications for the implementation of market strategies with regard to NFPs. First, it allows us to know the number of subjects interested in consuming the product and the factors that influence their propensity to buy it. Second, it allows us to analyse the distribution of prices that consumers are willing to pay (demand function) and therefore to correctly identify the sales price (given that the production costs are known). Identifying the relationship between the characteristics of the good, the decision to purchase it and the definition of the WTP also provides information on the characteristics that the good must have to be widely successful on the market. Through the model, it is therefore possible to have important information about the segmentation of the market, which is a fundamental factor for the success of an NFP.

In summary, our approach has the following original aspects: (i) it combines sensory preferences with a hypothetical market; (ii) it applies Cragg’s double-hurdle model to test which characteristics influence the decision to buy the product and then the stated WTP; and (iii) it is one of the few studies that applies the CVM and sensory analysis to a meat product.

## 2. Material and Methods

### 2.1. Data Collection

Data were collected during a sensory test by means of a computer-based questionnaire in the sensory analysis centre based in Matelica (Italy) in 2014. Overall, 252 participants were involved and completed the questionnaire. In three cases, the respondents did not declare their WTP; therefore, three questionnaires were discharged during our data analysis. The final sample was therefore 249 respondents. Participants were members of a panel of respondents from CIAS (Centro Italiano di Analisi Sensoriale), the Italian Centre for Sensory Analysis. The selection criteria required participants to be tinned meat consumers. CIAS uses trained panelists and verifies sensory panels at least once a year. Twenty-one sessions were carried out using an average of 12 panelists, a size that is considered optimal for a panel test [12]. Each session lasted aboutapproximately 90 min, of which 30 min were used for the initial training (description of the product and explanation of the questionnaire)and 60 min for the tasting test and completion of the questionnaire. Respondents’ socio-economic characteristics are presented in Table 1.

### 2.2. Study Structure

Our study combines two approaches. First, respondents were involved in a sensory test, and they then entered the economic valuation phase of the questionnaire used to estimate their WTP for the product (CVM hypothetical market).

#### 2.2.1. Sensory Test

During the first phase, respondents received a description of the product (tinned Chianina meat) they were going to taste (kept general to avoid influencing the respondents). After tasting the product, they were asked to judge their sensory experience regarding five aspects: (i) appearance; (ii) smell; (iii) flavour; (iv) texture; and (v) overall liking. For each aspect, the participants expressed their degree of liking on a 9-point hedonic scale [13], from 1 (I dislike extremely) to 9 (I like extremely). Such a hedonic scale was utilized because this type of test is specific to ask consumers to express their preference for food and score how much they like it [12,14,15].

The product was stored in a dedicated refrigerator at a controlled temperature between 1 °C and 4 °C before being prepared for sensory testing. During the test, the product was served on small disposable plastic plates with lettuce leaves so as not to affect the meat monochromaticity and was presented in test rooms complying with established standards (ISO 8589—Sensory Analysis—General Guidance for the Design of Test Rooms) [16]. Lighting, temperature, and relative humidity in the sensory analysis room were optimized and monitored to avoid any conditioning resulting from the experimental context.

#### 2.2.2. Hypothetical Market

With regard to the second phase, the economic valuation was performed applying the CVM [17] using a payment card elicitation format.

Respondents were first asked whether they were willing to buy the product—a 90 g tin of Chianina meat—they had just tasted and then, in the event of an affirmative answer, to indicate the maximum amount they were willing to pay for it, choosing from values ranging from € 1.20 to € 3.60 at incremental steps of € 0.20.

### 2.3. Methodological Background and Data Analysis

#### 2.3.1. The Contingent Valuation Method

The contingent valuation method, despite being developed in the ’60s by environmental economists [18,19] to valuate of public goods and gaining popularity during the ’80s [20], saw several applications in agribusiness marketing. Concerning its application for the valuation of consumers WTP for food, according to [21], it is possible to find 8 studies [22,23,24,25,26,27] that applied such methodology in conjunction with product tasting for the analysis of meat products (beef and pork), and in the majority of cases (6 out of 8) for beef.

The CVM method is part of stated preference methods and data are collected by means of questionnaires. In a typical CVM questionnaire developed for market goods, respondents are presented a hypothetical purchase scenario, where the good under valuation is presented and carefully described. In our study, the hypothetical purchase scenario included a description of the product that the respondents were tasting during the sensory test. To estimate consumers WTP for the product presented, a specific question usually follows the hypothetical purchase scenario in which respondents are requested to declare their WTP for the proposed product (a zero WTP declared implies that the respondent does not want to buy the product). Different WTP elicitation formats can be used (open-ended, payment card, dichotomous choice); in our study we opted for payment card elicitation format given that it tends to do not overestimate WTP.

As is known, the CVM approach presents many potential biases; among them, the so-called “hypothetical bias” is of particular relevance [20]. Many studies have highlighted that the CVM generally overestimates the true value of goods. However, by means of a meta-analysis, List and Gallet [28] and Murphy et al. [29] found that overestimation is less severe in the case of private goods than in the case of public goods. In a subsequent study, Tempesta [30] found that the ratio between hypothetical and real WTP tends to increase with the WTP amount; thus, in the case of tinned meat, which has a low price, the discrepancy between real and hypothetical WTPs may not be relevant. In this respect, one should also consider that the WTP estimated by means of a payment card elicitation format is usually lower than that estimated with other closed-ended formats [31,32,33].

The WTP is typically estimated by considering the mean or median WTP declared by respondents and the median is usually considered more conservative. Different statistical models can then be applied to better understand the determinants of the declared WTP.

#### 2.3.2. The Cragg’s Double-Hurdle Model

The hypothesis that drives our data analysis is that consumers’ decision processes can be divided into two steps [10]: first, consumers decide whether they are interested in buying the proposed product, and then, if they are willing to buy it, they decide the amount they are willing to pay for the product. According to Cragg [10], these two decision phases can be defined as participation decisions and consumption (sometimes referred to as outcome or quantity) decisions, respectively. In our case, the participation choice implies that the respondents stated that they were not willing to buy the product or declared a WTP = 0, while the consumption decision relates to the magnitude of the declared WTP. Two problems then become relevant: first, to understand the determinants that characterise those who are keen to enter the Chianina tin market and then to understand what determines a higher or lower declared WTP.

The results of Cragg’s approach are particularly useful in our case given that the cosumer’s decision not to buy the proposed good might result in a high frequency of corner solutions, particularly of zero-WTP observed data. Cragg’s double-hurdle model analyses the participation decision (first hurdle) by applying a probit model and then a truncated regression to study the consumption decision (second hurdle). The advantage of the Cragg [10] approach, compared to the Tobit model [34]—which is popular in the analysis of outcomes that pile up to zero—is that different independent variables can be used in the two hurdles. Even if the same variables are used, it allows them to have different coefficients in terms of magnitude and sign. In this respect, the same variable can have a negative impact on the participation hurdle and a positive or no impact on explaining the consumption decision.

Specifically, the participation equation can be written as:

(1) $$\begin{array}{cc} D & =\left\{\begin{array}{cc} 1 & \mathrm{if}\;\mathrm{WTP}\;>0\\ 0 & \mathrm{if}\;\mathrm{WTP}\leq 0 \end{array}\right. \end{array}$$

(2) $$\begin{array}{cc} D & =\alpha^{\prime }Z_{i}+u_{i} \end{array}$$

where *D* is a latent participation variable that takes value 1 if the declared WTP is greater than 0 and 0 otherwise; α is a vector of parameters; *Z* is a vector of respondent characteristics; and *u* is the error term.

The ‘consumption’ equation that allows us to derive the dependency of the stated WTP is:

(3) $$\begin{array}{cc} D & =1 \end{array}$$

(4) $$\begin{array}{cc} \mathrm{WTP} & =\beta^{\prime }X_{i}+v_{i} \end{array}$$

where WTP is the declared positive WTP; β is a vector of parameters; *X* is a vector of respondent characteristics; and *v* is the error term.

The dependent variable in the consumption equation assumed to be linear in Equation (4) is often modelled as logarithmic Equation (5):

(5) $$ln\left(\mathrm{WTP}\right)=\beta^{\prime }X_{i}+v_{i}$$

By exponentiating both sides of Equation (5), it is then possible to obtain Equation (6):

(6) $$\mathrm{WTP}=exp(\beta^{\prime }X_{i}+v_{i})$$

If the consumption decision is modelled using Equation (6), an exponential model is estimated, while the model is linear with Equation (4).

We analysed our data using Stata software (version 16) [35], particularly the churdle Stata 16 module. We estimated two models, one linear and one exponential.

In our models, we considered four categories of attributes:

1. the hedonic scores expressed during the sensory test;
2. the frequency of purchase of tinned meat;
3. socio-economic characteristics; and
4. the respondents’ opinions regarding Chianina meat.

Regarding the sensory preferences, the hedonic scores relating to flavour, smell and texture were included in the model. Due to multicollinearity problems, we decided to exclude the score relating to appearance. The choice to exclude this attribute derives from the fact that, in the purchase of tinned meat, the appearance is certainly less relevant than in the case of meat or other food products purchased fresh.

With reference to the frequency of purchase, a dummy variable (LowTin) equal to 1 was inserted for those who purchase less than 3 kg of tinned meat per year.

Regarding socioeconomic characteristics, the model included four age-related dummy variables (age: 31–40; age: 41–50; age: 51–60; and age: ≥60), a dummy variable for gender (male = 1) and three dummy variables related to educational level (lower secondary school, upper secondary school, college degree).

Finally, to account for their opinions on Chianina meat, respondents were asked to indicate with a score from 1 to 5 their degree of agreement with each of the following statements (where 1 = I don’t agree and 5 = I totally agree):

1. I am buying a healthy product;
2. I am buying a product bred according to traditional techniques;
3. I am buying an organic product;
4. I am buying a local product for which the distribution chain is short;
5. I am helping to preserve the traditional agricultural landscape;
6. I am helping to preserve biodiversity;
7. I am helping to protect animal welfare; and
8. I am buying a product guaranteed by the protected designation of origin (PDO)/protected geographical indication (PGI) European Union quality labels.

A factor analysis [36] was applied to reduce the number of variables to be included in the model to consider respondents’ opinions on Chianina meat. The varimax rotation procedure was used to identify the factors; in the extraction procedure, we considered the factors with an eigenvalue greater than or equal to 1. Two factors were then identified that could explain 66.4% of the total variance. The first factor can be defined as environmental quality (Table 2), while the second mainly concerns opinions relating to the intrinsic quality of the product.

## 3. Results

### 3.1. Sensory Preferences

Considering the five dimensions of the hedonic scales used during the sensory test, it is interesting to analyse the results considering the full sample first and then divide the sample into two groups: those who were willing to buy the product (WTP > 0, 88 respondents, 35% of the sample) and those who were not (161 respondents, 75% of the sample) (Table 3).

In the full sample (Table 3), respondents had a moderate overall appreciation of the product (mean rating on overall liking = 5.33), the most appreciated sensory dimension was flavour (mean rating flavour = 5.73), and the least appreciated dimension was texture (mean rating texture = 4.81).

Regarding the differences between respondents who were willing and not willing to buy the product (Table 3), the mean ratings of the two groups are significantly different (Table 4) and the respondents not willing to buy the product had a lower mean rating on all four sensory aspects than those willing to buy the product. For all aspects, the mean rating of people not willing to buy the product is always lower than 5, while the opposite is the case for those willing to buy the product. The aspects with the greatest difference in ratings between the two groups are smell and flavour, with a difference of 2.08 mean rating points in both cases (4.22 vs. 6.30 for smell, 4.99 vs. 7.07 for flavour). Interestingly, the two groups are consistent in terms of indicating the hierarchy of the sensory characteristic of the product. The aspect that had the highest rating for both groups is flavour, followed by smell and texture.

**Figure 1:**
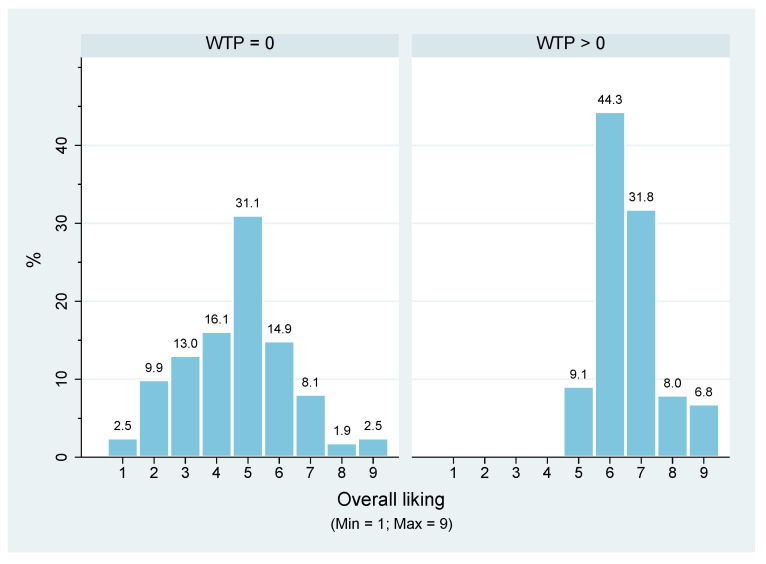
Overall liking rating of respondents (N.subjects = 249) by WTP declared (group 1—161 respondents: WTP = 0; group 2—88 respondents: WTP > 0). The participants expressed their degree of overall liking on a 9-point hedonic scale, from 1 (I dislike extremely) to 9 (I like extremely).

In Figure 1, it is evident that the overall liking rating differed between the two groups; interestingly, those respondents willing to buy the product always gave a rating on their overall liking greater than 4.

One interesting aspect is that some respondents (27.33%) gave an overall liking rating greater than 6 despite not being willing to purchase the product (WTP = 0) (Figure 1). Among them, approximately 14% gave a rating of 6, and approximately 8% gave a rating of 7. This behaviour is difficult to interpret, and we suppose that this can be explained by the respondents liking the tinned meat they were usually buying more than the tinned meat tasted in the experiment. Therefore, although they appreciated how the tinned Chianina meat tasted, they were not willing to buy it because they preferred the tinned meat they were accustomed to buying.

### 3.2. Contingent Valuation Results

**Figure 2:**
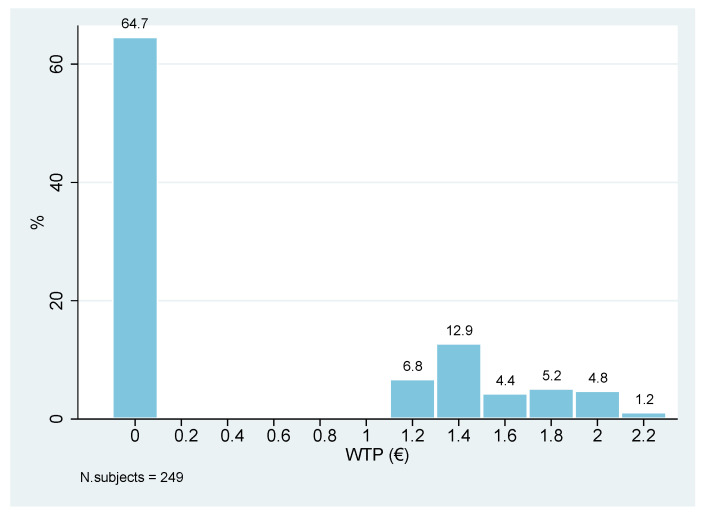
Contingent valuation results: histogram of declared WTP for a 90 g tin of Chianina meat by respondents (full sample). Percentages calculated on 249 subjects.

**Figure 3:**
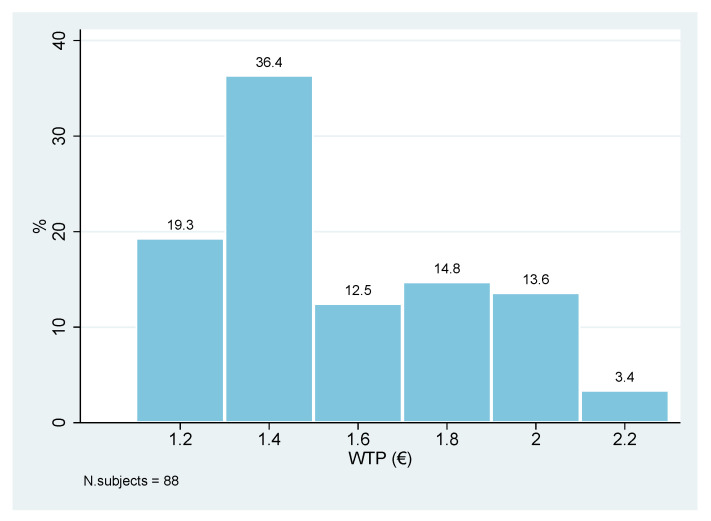
Contingent valuation results: histogram of declared WTP for a 90 g tin of Chianina meat by respondents with a positive WTP. Percentages calculated on 88 subjects.

Regarding the valuation of the proposed NFP, 35% of the sample (88 respondents) expressed a positive WTP, while the remaining 75% (161 respondents) declared a null WTP (Figure 2). Those willing to buy the NFP expressed a mean WTP of € 1.56 (Standard Error = 0.031; 95% Confidence Interval [1.49, 1.62]) and a median WTP of € 1.4 (Figure 3).

#### WTP Determinants

To understand the determinants of the expressed WTP in the CVM question, two double-hurdle models were estimated using the Stata 16 [35] churdle module (an alternative Stata module that could be used for the estimation of double-hurdle models is the craggit module [37]): the first is considered a linear consumption equation (Lin-MOD), while the second is considered an exponential one (Exp-MOD) (exponential models are sometimes referred to as lognormal when using other statistical software, such as the R [38] package mhurdle (version 1.1-8) [39]). The results of the estimated models are presented in Table 5, and the estimated coefficients of the participation models do not change between the two models because they share the same equation (Equation (2)). The two specifications of the consumption equation (linear or exponential) of the double-hurdle model do not differ in terms of the significance of the parameters, but the exponential specification is preferable for predictive purposes according to the AIC, BIC and $R^{2}$ statistical indices. In this respect, we will only comment on the exponential model results.

The participation results of the double-hurdle model (Exp-MOD) highlight that all coefficients of the sensory variables are statistically significant and have a positive sign: the higher the hedonic score given by respondents in terms of smell, flavour and texture, the higher the probability of purchasing the NFP. In terms of magnitude, the sensory coefficient that has the greatest impact is smell, followed by flavour and texture. Among the socio-economic and attitudinal variables, the only significant coefficients are LowTin and belonging to Factor 2 (product quality—related to factor analysis of respondents’ opinions on Chianina meat). Specifically, both coefficients have a negative sign, which implies that respondents who usually buy less than 3 kg of tinned meat per year and respondents who belong to Factor 2 (product quality) have a lower probability of entering the market for the tinned Chianina meat tasted.

Regarding the consumption model, the sensory coefficients still play a crucial role: they are all statistically significant. The only statistically significant coefficient among the socio-economic variables is gender (male), which has a positive sign, indicating that male respondents tend to be willing to pay more once entering the NFP market.

**Figure 4:**
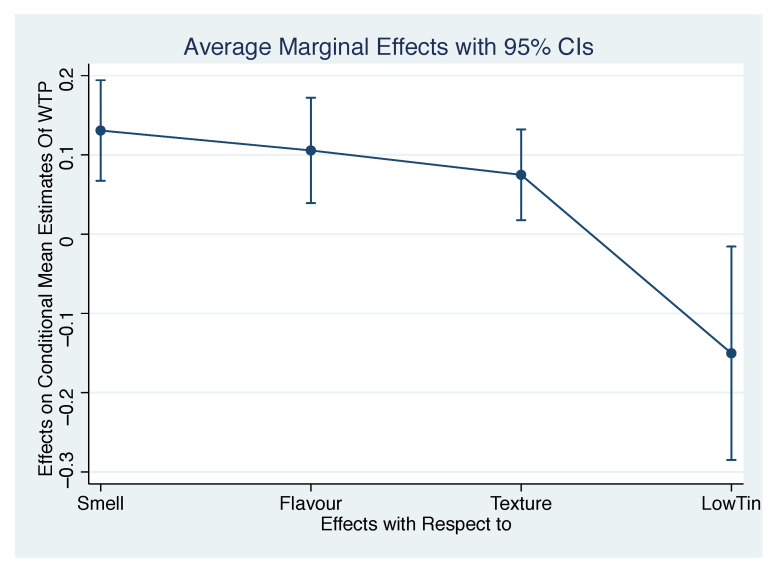
Overall liking and WTP of respondents with a WTP > 0 (88 respondents on 249 subjects).

To better understand the effect on the WTP of the variables considered, we analysed the average marginal effect (AME) on conditional mean estimates (see column 3 of Table 5 and Figure 4) of the dependent variable WTP of the quantity model. Four variables have statistically significant marginal effects on the estimated WTP. The most impacting change in WTP is given by smell rating: a one-unit change in smell rating increases WTP on average by € 0.13. Flavour is second in order of magnitude: a one-unit change in flavour rating has an AME on WTP of € 0.10. Texture is also noticeable in terms of its AME on WTP, with a positive contribution of € 0.07 for each unit change in rating. A low consumption of tinned meat (LowTin) decreases WTP by € 0.15.

## 4. Discussion

The investigations that have analysed the effect of tasting on WTP are numerous (see Torquati et al. [21] for a review). Generally, however, in these studies, the WTP has been related to the overall liking and only in some cases to the individual components of liking [40,41,42,43,44,45,46,47,48,49]. In our research, we sought to verify whether smell, flavour and texture have a different effect on inducing consumers to buy the product. This information can be useful to better define the characteristics of the product before its launch on the market than the analysis of overall liking alone. On the other hand, few studies have used double-hurdle models to identify the factors influencing consumers’ WTP [50,51,52,53,54].

One finding that emerged from our study is that only a limited fraction of respondents (35%) are interested in buying tinned Chianina meat. This is probably because the product is not perceived as particularly innovative compared to other types of tinned meat already on the market, especially with regard to taste. This finding may explain why 27.3% of those who gave an overall liking rating greater than 6 are not willing to buy the product. It is possible that while liking the proposed NFP, consumers prefer the products they currently buy, which may have a better quality–price ratio. This result is particularly relevant since it highlights that the satisfaction expressed by tasting the product does not necessarily imply that the product will be purchased. Therefore, to obtain a better understanding of the chances of success of an NFP, it is appropriate to associate tasting with the WTP analysis.

For the participation model, the decision to purchase the product is greatly influenced by the hedonic evaluation (in particular by smell), but current purchasing habits and, to a lesser extent, opinions on purchasing play an important role. With regard to purchasing habits, consuming little tinned meat greatly reduces the likelihood that Chianina meat will be purchased by those who have expressed a rather low overall-liking rating, while it has a much lower effect among those who liked it a great deal.

Regarding opinions, an element of some interest is the fact that respondents who belong to Factor 2 (product quality) have a lower probability of purchasing the NFP under analysis. Those who consider Chianina a high-quality meat with regard to health, the environment and the protection of traditional Italian products are less likely to buy it tinned. Probably for consumers, this type of food processing may considerably reduces many of the positive aspects associated with Chianina meat.

Unlike the findings in other studies that have used double-hurdle models to analyze consumers’ WTP [50,51,52,53,54], the socio-economic characteristics of the respondents do not seem to affect the probability of purchase.

A further aspect of importance, however, emerged in other studies [50,51,52,53,54], in that the factors that influence the probability of purchase are in part different from those that influence the WTP. A possible explanation for this observation is that the choice to buy the product depends more on liking and therefore on emotional factors that operate at an unconscious level (unconscious learning). To establish the WTP, the consumer instead resorts to more rational cognitive processes based on comparisons with the price of similar goods (substitutes). However, in this case, the consumer also often makes use of heuristics that simplify the decision-making process. However, our research showed that the different liking components seem to influence the probability of purchase and WTP in different ways. In particular, we observed that the choice to buy the product seems to be greatly influenced by smell and, to a much lesser extent, by texture. WTP, on the other hand, is particularly influenced by texture and less by smell.

Although many studies have found that taste/flavour had the greatest impact on WTP ([41]—apples; [42]—beer; [44]—wine; [46]—ciders; [48]—beer), other research has highlighted that, as in our study, other factors may be also important ([40]—fruits; [43]—apples; [45]—apples; [49]—wine).

## 5. Conclusions

Including sensory dimensions in the analysis of consumers’ WTP for an NFP disclosed some interesting insights for the future application of the CVM method in the context of agribusiness marketing. Our analysis highlighted that sensory hedonic scores play a crucial role in determining both participation in the market and the magnitude of WTP compared to the other socio-economic and attitudinal variables.

Regarding the first objective of our research, our results show that all sensory preferences considered influenced consumer propensity to buy the product, while only two attitudinal variables (quantity of tinned meat bought per year and opinions—Factor 2/product quality) influenced this aspect. In particular, the higher the hedonic score given by the respondent for flavour, smell and texture, the higher the probability that he/she is willing to buy it.

Regarding the second objective of our research, we can conclude that sensory preferences are the only factors among those considered, apart from gender, that affected the stated WTP. The analysis of the AME on conditional mean estimates showed that, for our product, the rating on smell has the greatest impact on WTP (a one-unit change in smell rating increases WTP on average by € 0.13), followed by flavour (€ 0.10) and texture (€ 0.07).

Regarding the third objective, we found that the sensory aspects, despite being crucial in both the participation and consumption models, play different roles in these two phases. While smell and flavour are the most important aspects in determining the probability of entering the market, texture is the sensory variable that has the greatest impact among the sensory variables in the consumption model (namely, on the WTP). The latter result is confirmed by both the linear and exponential specifications of the consumption model, where the texture coefficient is greater than that of flavour and smell, respectively.

Our results therefore appear to confirm those obtained in the few previous joint applications of CVM and sensory tests, where other authors [41,42,45,47] found a positive relationship between sensory rating and WTP. However, those authors studied fruit products, while we applied a combined approach to a new meat product in our study.

In conclusion, these results seem to highlight that in defining the characteristics of an NFP that influence consumers’ propensity to buy the product, it is probably more appropriate to consider the different components of liking and not only overall liking. For those who develop an NFP, it is necessary to know which aspects of liking most contribute to its success once it is placed on the market. As noted in the Discussion, it is also important that hedonic evaluations are accompanied by economic valuations since a high degree of liking does not necessarily imply a willingness to purchase the product.

Further research should be carried out to confirm the existence of an effective diversity between factors that influence the propensity to buy and the factors that underlie the WTP for an NFP, since this could lead to interesting indications for the definition of marketing strategies for the launch of NFPs on the market.

## Figures and Tables

**Table 1:** Socio-economic characteristics of respondents.

	No.	%
Gender		
Female	158	63.5
Male	91	36.5
Total	249	100.0
Age		
≤30	70	28.1
31–40	61	24.5
41–50	49	19.7
51–60	49	19.7
≥60	20	8.0
Total	249	100.0
Education level		
Middle school	23	9.2
High school	130	52.2
Graduate	96	38.6
Total	249	100.0
Household size		
1–2	103	41.4
3–4	122	49.0
≥5	24	9.6
Total	249	100.0
Household income (gross annual)		
No information	80	32.1
≤10,000 €	38	15.3
10,001–30,000 €	89	35.7
30,001–50,000 €	34	13.7
>50,000 €	8	3.2
Total	249	100.0
In charge of household purchases		
No	31	12.4
Yes	107	43.0
Yes, with another family member	111	44.6
Total	249	100.0

**Table 2:** Factor analysis of respondents’ opinions on Chianina meat: the rotated component matrix.

Statements	Factor 1	Factor 2
	Environmental Quality	Product Quality
I am buying a healthy product	0.2119	0.7427
I am buying a product bred according to traditional techniques	0.0873	0.8931
I am buying an organic product	0.2542	0.6941
I am buying a local product for which the distribution chain is shortening	0.4670	0.6317
I am helping to preserve the traditional agricultural landscape	0.7601	0.3592
I am helping to conserve biodiversity	0.8834	0.1669
I am helping to protect animal welfare	0.8778	0.1545
I am buying a product guaranteed by the brand (PDO/GPI)	0.6250	0.2153

**Table 3:** Summary statistics of sensory hedonic scores *.

Sensory Aspect	Sample	No.	Mean	SD	Median	Min	Max
Overall liking	Full Sample	249	5.33	1.76	5	1	9
	WTP = 0	161	4.65	1.70	5	1	9
	WTP > 0	88	6.59	1.00	6	5	9
Flavour	Full Sample	249	5.73	1.91	6	1	9
	WTP = 0	161	4.99	1.85	5	1	9
	WTP > 0	88	7.07	1.14	7	4	9
Smell	Full Sample	249	4.95	1.87	5	1	9
	WTP = 0	161	4.22	1.78	4	1	8
	WTP > 0	88	6.30	1.16	6	4	9
Texture	Full Sample	249	4.81	1.79	5	1	9
	WTP = 0	161	4.22	1.75	4	1	8
	WTP > 0	88	5.89	1.33	6	4	9

* Hedonic scores collected using a 9-point likert hedonic scale for each of the aspects considered. The scores ranged from 1 (I dislike extremely) to 9 (I like extremely).

**Table 4:** Two-sample T-test with unequal variances: differences in mean hedonic scores between groups (group 1—161 respondents: WTP = 0; group 2—88 respondents: WTP > 0).

	Means by Group			
	**WTP** =0	**WTP** >0		**diff**
	$\mu_{1}$	$\mu_{2}$	***t***	$\mu_{1}-\mu_{2}$
Overall liking	4.65	6.59	−11.33	−1.94 ***
Flavour	4.99	7.07	−10.93	−2.07 ***
Smell	4.22	6.30	−11.14	−2.08 ***
Texture	4.22	5.89	−8.43	−1.66 ***

*** *p* < 0.001.

**Table 5:** Double-hurdle model results.

	Lin-MOD		Exp-MOD		AME Exp-MOD	
**Participation**	β	SE	β	SE	β	SE
Smell	0.343 ***	(0.10)	0.343 ***	(0.10)		
Flavour	0.258 *	(0.11)	0.258 *	(0.11)		
Texture	0.159 +	(0.09)	0.159 +	(0.09)		
LowTin ${}^{†}$	−0.545 *	(0.22)	−0.545 *	(0.22)		
Age 31–40	0.138	(0.29)	0.138	(0.29)		
Age 41–50	0.154	(0.32)	0.154	(0.32)		
Age 51–60	−0.244	(0.33)	−0.244	(0.33)		
Age ≥60	−0.118	(0.48)	−0.118	(0.48)		
Gender = Male	−0.049	(0.22)	−0.049	(0.22)		
Education Middle school	0.483	(0.40)	0.483	(0.40)		
Education High School	−0.023	(0.47)	−0.023	(0.47)		
Education Graduate	0.519	(0.46)	0.519	(0.46)		
Factor 1	0.091	(0.11)	0.091	(0.11)		
Factor 2	−0.214 +	(0.13)	−0.214 +	(0.13)		
Constant	−4.720 ***	(0.78)	−4.720 ***	(0.78)		
**Consumption (WTP)**						
Smell	0.063 *	(0.03)	0.038 *	(0.02)	0.131 ***	(0.03)
Flavour	0.064 *	(0.03)	0.042 *	(0.02)	0.106**	(0.03)
Texture	0.072 ***	(0.02)	0.044 ***	(0.01)	0.075 *	(0.03)
LowTin ${}^{†}$	0.060	(0.05)	0.043	(0.03)	−0.150 *	(0.07)
Age 31–40	−0.002	(0.06)	−0.001	(0.04)	0.044	(0.10)
Age 41–50	−0.029	(0.07)	−0.022	(0.05)	0.037	(0.11)
Age 51–60	0.059	(0.08)	0.038	(0.05)	−0.060	(0.11)
Age ≥60	−0.178	(0.11)	−0.105	(0.07)	−0.089	(0.14)
Gender=Male	0.082 +	(0.05)	0.051 +	(0.03)	0.012	(0.07)
Education Middle school	0.044	(0.11)	0.021	(0.07)	0.164	(0.13)
Education High school	0.022	(0.13)	−0.000	(0.08)	−0.007	(0.14)
Education Graduate	0.031	(0.12)	0.008	(0.08)	0.168	(0.15)
Factor 1	0.027	(0.03)	0.016	(0.02)	0.038	(0.04)
Factor 2	0.010	(0.03)	0.004	(0.02)	−0.066	(0.04)
Constant	0.203	(0.20)	−0.414 **	(0.13)		
lnsigma						
Constant	−1.546 ***	(0.08)	−2.009 ***	(0.08)		
N. respondents	249		249			
LogLikelihood	−87.75		−3.51			
$R^{2}$	0.51		0.96			
Chi${}^{2}$	179.10		178.19			
AIC	237.49		69.01			
BIC	346.53		178.05			

Standard errors in parentheses. † A dummy variable indicating respondents who purchase less than 3 kg of tinned meat per year. Significance levels: + *p* < 0.10, * *p* < 0.05, ** *p* < 0.01, *** *p* < 0.001.

## Data Availability

The data that support the findings of this study are available on request from the corresponding author (D.V.).

## Abbreviations

The following abbreviations are used in this manuscript:

AME	average marginal effect on conditional mean estimates
CVM	contingent valuation
DCE	discrete choice experiment
Exp-MOD	exponential model
Lin-MOD	linear model
LowTin	dummy variable equal to 1 for those who purchase less than 3 kg of tinned meat
	per year
NFP	new food product
WTP	willingness to pay

## Appendix A

Chianina meat derives from the Chianina breed of cattle, a perfect animal from a morphological point of view and made unique by the somatic gigantism, the length of the trunk and the white porcelain coat. Chianina meat has undisputed bromatological characteristics and it is commercially very appealing thanks to high standards of quality, traceability and healthiness. The breeding of Chianina cattle is part of the zootechnical tradition of central Italy and the meat produced represents a typical product of excellence strongly linked to the production area, of which it incorporates distinctive and unrepeatable characteristics.

Like many products of excellence, the enhancement process took place through the creation of a specification and a quality logo (Protected Geographical Indication (PGI) “Vitellone Bianco dell’Appennino Centrale”—The Protected Geographical Indication label is part of the European Union quality logos—Regulation (EU) No 1151/2012) and the construction of social and market relationships between actors involved.
